# Quality of life associated with immunosuppressant treatment adherence in liver transplant recipients: A cross-sectional study

**DOI:** 10.3389/fphar.2023.1051350

**Published:** 2023-02-24

**Authors:** Mojtaba Shafiekhani, Farbod Shahabinezhad, Zahra Tavakoli, Tahereh Tarakmeh, Elham Haem, Negar Sari, Shohreh Nasirabadi, Masoud Dehghani

**Affiliations:** ^1^ Shiraz Transplant Research Center, Shiraz University of Medical Sciences, Shiraz, Iran; ^2^ Shiraz Transplant Center, Abu-Ali Sina Hospital, Shiraz University of Medical Sciences, Shiraz, Iran; ^3^ Department of Clinical Pharmacy, Faculty of Pharmacy, Shiraz University of Medical Sciences, Shiraz, Iran; ^4^ Department of Biostatistics, School of Medicine, Shiraz University of Medical Sciences, Shiraz, Iran

**Keywords:** solid organ transplantation, quality of life, adherence, immunosuppressive medication, liver transplantation

## Abstract

**Purpose:** Transplanted organ survival after solid organ transplantation highly correlates with the adherence levels of the patients to their immunosuppressive drugs. On the other hand, one of the main goals of liver transplantation is to increase the overall quality of life (QOL) for the patient. This study is aimed to analyze the relationship between adherence and QOL in adult liver transplant recipients of the biggest liver transplant center in Asia.

**Methods:** All of the included patients were older than 18 years and at least 6 months had passed from their liver transplantation. The adherence level was measured with BAASIS method and the QOL was assessed by SF-36 questionnaire in real-time interviews. The relationship between adherence and different aspects of QOL in addition to qualitative and quantitative influential factors on these two outcomes was calculated with statistical analysis.

**Results:** Among the 122 included patients, 41% of the were categorized in the non-adherent group. The most important reasons for non-adherence in these patients included forgetfulness (62%), lack of medication (12%), financial problems for drug supply (9%), and side effects (2%). According to the results of the multivariate linear regression model, rejection was the only influential factor in the occurrence of non-adherence among patients (OR = 8.226 CI (1.404-48.196)). The overall mean QOL score of patients was reported 51.09 ± 21.86. The lowest is given to social functioning, while mental health has achieved the highest score. The mean QOL scores in different dimensions in patients with adherence were higher than non-adherents, which was only significant in mental health (*p*-value = 0.01). Additionally, in total scores related to Physical Composite Score (PSC) and Mental Composite Score (MCS), the mean MSC scores in adherent patients were significantly higher than non-adherent patients (*p*-value: 0.02). Although adherent patients have an overall greater QOL, the only meaningful effect on QOL total score was from income level.

**Conclusion:** The overall QoL score has been in all parameters higher in the adherent group compared with non-adherent patients. The difference in QoL was most meaningfully significant in mental composite score among other parameters of QoL.

## Background

Liver transplantation is a lifesaving intervention available for patients suffering from end-stage liver diseases. There is an increasing number of liver transplant centers that provide liver transplant surgeries across the world. According to United Network for Organ Sharing (UNOS), 8,906 liver transplants were performed in the United States in 2020 (Organ transplant trends | More transplants than ever UNOS, 2021). Shiraz Transplant Center is the leader in solid organ transplantation in the Middle East and Asia and one of the largest liver transplant centers in the world, with more than 500 solid organ transplants per year (Malek-Hosseini et al., 2019). The first liver transplantation in Iran was performed in 1992 at Namazee Hospital, Shiraz. Since then more than 6,500 cases of liver transplants have been performed in Iran.

Immunosuppressive medications are still considered a critical component in patients’ post-transplant care. Adherence to an immunosuppressive regimen after liver transplantation guarantees graft survival (Jones and Serper, 2020). Non-adherence is one of the three primary causes of post-transplant organ failure, along with rejection and infection (Moradi et al., 2019). Treatment adherence is defined by the World Health Organization (WHO) as “the extent to which a patient’s behavior coincides with the clinical prescriptions” (Ahmed and Aslani, 2014).

The prevalence of non-adherence in solid organ transplant recipients has been estimated to be between 15% and 30% in various studies (Mehta et al., 2017). Non-adherence increases the risk for graft loss and post-transplant hospitalizations by about 25% (Takemoto et al., 2007). According to WHO the economic impact of non-adherence in solid organ transplant recipients is estimated to be between 15 and 100 million USD annually (Gorevski et al., 2013). Albekairy et al. Reported about 41% of adult liver transplant recipients were non-adherent to their immunosuppressive treatment (Albekairy et al., 2016).

Different adherence levels measurement method has resulted in a wide range of non-adherence reports. Factors that affect a patient’s non-adherence to their treatment have been mentioned in different studies, including age at the time of transplantation, underlying diseases, education level, marital status, employment status, and income (Mehta et al., 2017; Jones and Serper, 2020).

An important goal after transplantation is to increase the quality of life (QoL) in recipients. WHO defines QoL as “an overall sense of wellbeing, including aspects of happiness and satisfaction with life as a whole**”.** It includes factors such as mental, physical, and social status (Butt et al., 2012; Burra et al., 2018). Several studies have been published on the relationship between liver transplants and factors affecting QoL in them. According to the studies, male gender, model for end-stage liver disease (MELD) score before transplantation, having a supportive family, and marriage are among the factors that improve QoL after liver transplant (Burra et al., 2018; Jones and Serper, 2020). QoL is also measured using questionnaires. One of the well-known questionnaires is the Short Form 36 (SF-36), which measures different physical, mental, social, and pain factors in varied conditions (Ware, 2000). No studies have been written on the relationship between immunosuppressive medication adherence and QoL in adult liver transplant recipients in Iran. Therefore, the goal of this study has been to evaluate the level of adherence and QoL and the association between these two outcomes in adult liver transplant patients of Shiraz Transplant Center as one of the world’s greatest solid organ transplant centers.

### Study design

This is a cross-sectional study conducted at the Abu-Ali Sina Transplant Hospital in Shiraz, Iran, affiliated to Shiraz University of Medical Sciences, Iran, from April 2020 to February 2021. The study has been approved by the Shiraz University of Medical Sciences Institutional Review Board (#IR.SUMS.REC.1399.494).

### Patient enrolment

**FIGURE 1:**
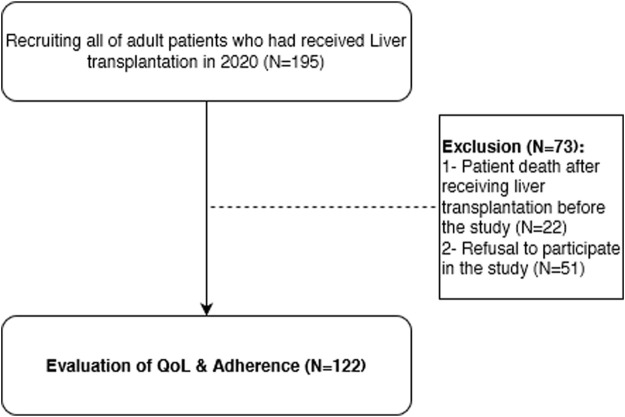
Patient enrolmentin and follow up during the study (N = 122).

We enrolled liver transplant recipients over the age of 18 years, who received at least one immunosuppressive drug treatment and at least 6 months have been passed after their transplantation. Patients who had received other solid organ transplantation in addition to their liver transplant or went through liver re-transplantation were excluded from the study. The considered 6-month period was to ensure achieving a stable level of immunosuppressive medications as well as stabilizing the patient’s clinical condition. Patient enrolment is briefly described in Figure 1.

### Data collection

We collected and recorded socio-demographic data including age, gender, level of education, employment, economic and marital status and living condition, clinical data including the cause of liver failure, donor type, duration of liver transplantation, comorbidities, underlying diseases, and acute rejection episodes, habitual history data including smoking and drug or alcohol consumption and immunosuppressive regimen and other medications history. These data and evaluation of adherence level and QoL were collected from face-to-face interviews, as well as reviewing medical records stored in the hospital database by two registered nurses trained in the field of transplantation under the supervision of a clinical pharmacist and transplant surgeon.

### Adherence evaluation

Adherence level to immunosuppressive medications was assessed in face-to-face interviews using the Basel Assessment of Adherence to Immunosuppressive medication Scale criteria (BAASIS) (Dobbels et al., 2010). This criterion consists of four items related to the use of immunosuppressive drugs such as dimension, timing (drug holiday), dose reduction, and continuity of immunosuppressive medication administration. Responses were given on a six-point scale: every day 5), more than once per week 4), every week 3), every second week 2), once per month 1), and never 0). Any answer other than “never” in each of the four questions was considered non-adherent. It was also recorded if a patient used reminders to take their medications, such as timed pillboxes or calendars.

### Quality of life assessment

The quality of life (QoL) level was measured by the short-form (SF-36) questionnaire and was confirmed by face-to-face observations made by trained interviewers when the adherence levels were accessed. SF-36 is created and validated as a standardized short-form instrument for measuring health-related QoL and is commonly used in the Medical Outcomes Study to measure essential QoL domains. The SF-36 consists of eight QoL domains: PF, physical functioning (10 questions); RP, role physical (4 questions); BP, bodily pain (2 questions); GH, general health (5 questions); VT, vitality (5 questions); SF, social functioning (2 questions); RE, role emotional (3 questions); and MH, mental health (5 questions) (Lins and Carvalho, 2016).

The standardized scores for each domain range from 0 to 100, where 0 reflects a diminished QoL. The eight scales are summarized in two scores: Physical Composite Score (PCS) for physical health, and Mental Composite Score (MCS) for mental health. The PCS score includes physical functioning, role physical, pain, and general health scales, and the MCS score comprises role emotion, social function, and scales emotional wellbeing scales. A Persian version has already been prepared and validated based on Iranian demographic norms (Motamed et al., 2005).

### Statistical analysis

We reported categorical data as a percentage and continuous variables as means ± standard deviation (SD). To perform the linear regression analysis with odds ratio (OR) and 95% confidence interval (CI), we used a stepwise method to determine the possible relation of different demographic, socio-economic, cultural, clinical factors, and development of acute rejection episodes with adherence. We selected variables with *p*-values less than 0.3 for the final multivariate logistic regression model, and we considered *p*-values less than 0.05 statistically significant. Also, to compare between adherent and non-adherent groups, the mean of answers for all questions and each SF-36 domain were calculated using the linear regression model. All the statistical analyses were performed by Social Science Package Statistical Software (SPSS) version16.

## Result

A total of 122 patients were included in the study. The mean age was 43.43 ± 13.57 years, and 70 (57.3%) of them were males. Table 1 shows the demographic, social-economical-cultural, and clinical data of the participants.

**TABLE 1:** Demographic, clinical, and social-economical-cultural properties of the liver transplant recipients (N = 122).

***Gender (%)***	
Female	43 (43)
Male	57 (57)
***Age***	
Mean ± SD	43.43 ± 13.57
***Age of Tx***	
Mean ± SD	40.40 ± 13.72
***Time since Tx*** (***%***)	
6–12 months	47 (38.52)
12–24 months	59 (48.36)
More than 24 months	16 (13.11)
***Donor type*** (***%***)	
Living donor	3 (2.5)
Deceased donor	119 (97.5)
***Cause of chronic liver disease*** (***%***)	
Cryptogenic cirrhosis	47 (38.52)
Hepatitis B virus	42 (34.43)
Autoimmune hepatitis	13 10.66)
Non-alcoholic steatohepatitis	11 (9.02)
Hepatocellular carcinoma	4 (3.28)
Hepatitis C virus	3 (2.46)
Others	2 (1.64)
***Comorbidity*** (***%***)	
Hypertension	29 (24.02)
Diabetic Mellitus	39 (32.11)
Dyslipidemia	15 (12.19)
CVD	28 (23.12)
Asthma and COPD	11 (9.10)
***Hx of Rejection*** (***%***)	
Yes	53 (43.10)
No	69 (56.90)
***Hx of hospitalization during 1 year ago*** (***%***)	
Yes	105 (86)
No	17 (14)
***Cause of hospitalization*** (***%***)	
Infection	63 (51.40)
Rejection	27 (22.14)
Biliary complications	15 (11.91)
PTLD	11 (8.71)
Others	7 (5.85)
***Waiting time for Tx*** (***%***)	
<1 year	101 (83)
>1 year	21 (17)
***Immunosuppressive regimen*** (***%***)	
CNI + Antimetabolites + Steroids	111 (91.10)
Antimetabolites + Steroids	7 (5.40)
CNI + Steroids	3 (2.72)
mTOR inhibitors + Steroids	1 (0.78)
***Employment status*** (***%***)	
Unemployed	46 (37.61)
Employed	29 (23.89)
Retired	22 (18.02)
Housekeeper	25 (20.48)
***Insurance*** (***%***)	
Insured	119 (97.6)
Uninsured	3 (2.4)
***Education*** (***%***)	
Illiterate	4 (3.31)
Less than 12 years	62 (50.81)
More than 12 years	56 (45.88)
***Marital status*** (***%***)	
Married	94 (77)
Single	28 (23)
***Living status*** (***%***)	
With family	120 (98.3)
Alone	2 (1.7)
***Opioid addiction*** (***%***)	
Never	118 (97.4)
Previously	2 (1.3)
Currently	2 (1.3)
***Alcohol consumption*** (***%***)	
Never	119 (97.2)
Previously	3 (2.8)
***Cigarette smoking*** (***%***)	
Never	114 (92.9)
Previously	4 (3.6)
Currently	4 (3.6)
***Monthly income*** (***USD***) (***%***)	
Lower than 1,000$/month	48 (39.2)
More than 1,000$/month	74 (60.8)
***Children*** (***%***)	
0-1	19 (15.3)
1-3	73 (60)
More than 3	31 (25.7)
***Distance to the Tx provider center*** (***%***)	
Less than 2 h	41 (34)
2–4 h	10 8)
More than 4 h	71 (58)
***Tacrolimus level***	
Mean ± SD	9.02 ± 7.40

Tx: transplant; COPD: chronic obstructive pulmonary disease; CVD: cardiovascular disease; PTLD: Post-transplant lymphoproliferative disease; CNI: calcineurin inhibitors.

The most common indications for liver transplantation were cryptogenic cirrhosis (38.52%), Hepatitis B (34.43%), and autoimmune hepatitis (10.66%). The most important comorbidities were diabetes mellitus (32.11%), hypertension (24.02%), and cardiovascular disease (23.12%). Of the included patients, 96.7% were literate, 23.89% were employed, while 98.3% lived with family and 1.7% lived alone.

History of alcohol or drug use was positive in 2.8% and 2.6% respectively. None of them did mention any continued use of alcohol during the study period, while 1.3% admitted being on opioid drugs in that period. Of the subjects of the study, 48.3% had participated in the study in 12–24 months and 13.11% in more than 2 years after their transplantation. In total, 43.1 percent of patients experienced at least one episode of rejection after liver transplantation. 91.10% of the participants received a triple immunosuppressive regimen including antimetabolite, Calcineurin inhibitor (CNI), and steroids. Among all patients, 41% (50 patients) were categorized in the non-adherent group. The most important reasons for non-adherence in these patients included forgetfulness (62%), lack of medication (12%), financial problems for drug supply (9%), and side effects (2%). The most common causes of drug holiday were:COVID-19 infection and discontinuation of several concurrent doses, in spite that there has been no presciptions for discontinuation. Forgetfulness and Travel and limited access to enough amounts of the medications.

Table 2 shows the relationship between quantitative and qualitative risk factors and the occurrence of non-adherence. Patients’ age, age of transplant, income level, history of hospitalization, and transplant rejection were among the main factors impacting non-adherence. Of those factors, according to the results of the multivariate linear regression model, rejection was the only influential factor in the occurrence of non-adherence among patients (OR = 8.226 CI(1.404-48.196)).

**TABLE 2:** Quantitative and qualitative risk factors regarding adherence among the liver transplant recipients (N = 122).

	OR (95% CI) [*p*-value] univariate	OR (95% CI) [*p*-value] multivariate
**Age**	0.978 (0.952–1.005) [0.111]	1.030 (−0.234-0.351) [0.797]
**Age of Tx**	0.975 (0.949–1.001) [0.063]	0.928 (−0.410 – 0.165) [.495]
**Rejection**	1.750 (0.659–4.648) [0.261]	5.241 (0.286–4.270) [0.009]
**History of hospitalization**	0.750 (0.273-2.682) [0.594]	0.552 (−22.367–20.810) [0.390]
**Sex**	0.67 (0.44-1.19) [0.66]	-
**Waiting time for Tx**	1.206 (0.435-3.347) [0.719]	-
**Occupation status**	0.640 (0.210-1.947) [0.431]	0.826 (−2.327-1.482) [0.824]
**Education**	1.465 (0.702-3.057) [0.600]	-
**Marital status**	0.646 (0.263–1.587) [0.341]	-
**Living condition**	1.0 (0.043-11.462) [1.000]	-
**Monthly income**	2.277 (0.737-7.030) [0.153]	2.971 (−0.728–3.616) [0.190]
**Distance to the Tx center**	1.071 (0.217-5.293) [0.933]	1.322 (−22.490-20.546) [0.560]

Tx: transplant; OR: odds ratio; CI: confidence interval.

The overall mean QoL score of patients was reported 51.09 ± 21.86. Accordingly, the lowest score is given to social functioning, while mental health has achieved the highest score (Table 3). The mean QoL scores in different dimensions in patients with adherence were higher than non-adherents, which was only significant in mental health (*p*-value = 0.01). Additionally, in total scores related to PSC and MCS, the mean MSC scores in adherent patients were significantly higher than non-adherent patients (*p*-value: 0.02).

**TABLE 3:** Different components of quality of life between adherent and non-adherent liver transplant recipients (N = 122).

*Component*	*Total (N = 122)*	*Adherent*	*Non-adherent*	*p-value*
	*Mean ± SD*	*N = 72*	*N = 50*	
***Physical functioning***	57.37 ± 26.51	58.29 ± 22.10	56.45 ± 30.92	0.72
***Social functioning***	44.01 ± 31.95	47.81 ± 35.55	40.22 ± 28.36	0.08
***General health***	61.39 ± 12.90	63.22 ± 14.42	62.56 ± 11.38	0.12
***Vitality***	51.21 ± 21.19	52.24 ± 19.26	50.18 ± 23.12	0.87
***Mental health***	61.83 ± 14.32	63.21 ± 15.59	58.25 ± 13.05	0.0 1
***Pain***	52.68 ± 30.06	54.27 ± 32.21	53.09 ± 27.91	0.44
***Role limitation*** (***physical***)	51.39 ± 31.00	53.34 ± 22.00	49.44 ± 40	0.09
***Role limitation*** (***emotional***)	49.76 ± 20.90	50.05 ± 21.21	49.4 ± 20.59	0.11
PCS*	55.83 ± 25.11	57.28 ± 22.68	55.38 ± 27.57	0.77
MCS**	48.32 ± 18.01	50.03 ± 25.34	46.61 ± 24.03	0.02

*PCS: physical composite score, **MCS: mental composite score.

Based on the results of Table 4, which evaluated the relationship between quantitative and qualitative variables affecting the total score of QoL, the only meaningful effect on QoL total score was of income level. Although adherent patients have an overall greater QoL compared with the others, the differences are not statistically significant.

**TABLE 4:** Different demographic, socio-economic, cultural, and clinical factors concerning the quality of life among liver transplant recipients.

*Variables*	*Standardized Coefficients*	*p-value*
***Occupation***	−0.07	0.58
No jobs (Reference)		
Others		
***Education***	−0.17	0.22
Diploma		
Medical sciences		
Non-medical sciences (Reference)		
Others		
***Marital status***	−0.21	0.09
Single (Reference)		
Married		
***Hx of hospitalization***	−0.06	0.66
Yes (Reference)		
No		
***Rejection***	0.21	0.12
Yes (Reference)		
No		
***Adherence***	−0.08	0.53
Yes (Reference)		
No		
***Income***	−0.33	0.01
Lower than 1,000$/month (Reference)		
More than 1,000$/month		
***Time since transplantation***	−0.74	0.12
Less than 1 year		
More than 1 year		

Hx: History.

## Discussion

Adherence to immunosuppressive drugs is one of the most critical factors in the success of the organ transplant process. On the other hand, one of the most important issues, which is often one of the main goals of the transplant process, is to improve the QoL after organ transplantation. This study aimed to determine the relationship between patients’ adherence to immunosuppressive drugs and QoL levels after liver transplantation, following similar earlier on kidney transplantation (Ganjali et al., 2019; Moradi et al., 2019). The non-adherence rate in our study was 41%. In some other studies on liver transplant patients, different ranges such as 32%–50.8% have been reported for non-adherence levels (Serper et al., 2015; Leven et al., 2017). One of the most important factors explaining this wide range of reported levels is the different methods used for adherence evaluation. For example, in our study, the measurement was based on the BAASIS method, while in the study of Serper et al., the plasma level of Tacrolimus was used as an adherence criterion (Serper et al., 2015). Therefore, due to the difference in measurement methods, diverse studies are not easily compared. Moreover, to name another causing factor, the time elapsed from the transplantation is effective on adherence as well. It has been shown that the more time passes from the transplantation, the less is that particular patient’s adherence. Thus, considering the heterogeneity of diverse studies with different study group populations and times elapsed since the transplantations, this wide range is expectable.

Patient forgetfulness was the most important cause of non-adherence in our study. In addition, the lack of medications was another major cause of non-adherence. Noteworthy, in the study of Moradi et al. and Cossart et al., forgetfulness has been considered the most important cause of non-adherence as well (Cossart et al., 2019; Moradi et al., 2019). Due to the dependence of Iran’s pharmaceutical industry on imported raw materials to supply the pharmaceutical industry and the existence of international sanctions against trade with Iran in the past decade, there are shortages of immunosuppressive medications at times (Setayesh and Mackey, 2016), explaining the second cause of non-adherence among the patients in our study.

History of re-hospitalization, history of rejection, younger age at the time of transplantation, low income, and unemployment were among the risk factors affecting non-adherence in univariate analysis. Ultimately, the history of rejection is calculated to be the only factor influencing the incidence of non-adherence in our study. In some studies, ages less than 40 years, psychiatric disorders, immunosuppressive side effects, and having beliefs that medications were harmful have been among the risk factors for non-adherence (Ghods et al., 2003; Drent et al., 2009). In the study of Ghods et al. (Ghods et al., 2003), which examined the risk factors for non-compliance among kidney transplant patients, the number of transplant rejections was one of the factors affecting non-adherent incidence.

Our study’s average total QoL score was about 51, which is much lower than the average QoL score reported in developed countries among adult liver transplant recipients. Fredericks et al. reported a total QoL score of close to 60.3, also in a study by Masala et al., it was estimated to be 63 (Fredericks et al., 2008; Masala et al., 2012). Different methods for measuring QoL and the dissimilarity between the average time elapsed since the time of transplantation between diverse studies have led to an unalike range for the average QoL in distinct studies, making comparisons between these studies more difficult. In this regard, some studies believe that QoL of liver transplant patients has an upward trend in the first 6 months after transplantation. However, in long-term follow-ups (5 years and 10 years), the upward trend has stopped, and over time since the transplant, QoL takes a decreasing trend (Telles-Correia et al., 2009; Burra and Germani, 2013). In conclusion, the factor of time elapsed after transplantation can be an essential influencer in comparing QoL measurements between different studies.

Based on our data, patients with poor psychological scores in the study period were the most non-adherent. However, there is no visible cause and effect association in this manner. In other words, it is still unclear whether non-adherence has caused a lower score in psychological factors, or it is psychological factors affecting the trend of patients to non-adherence. It is also possible for these factors to induce a positive loop for each other, as if, non-adherent patients will become less emotionally stable, which might cause more non-adherence in this population. Additionally, as mentioned earlier, there are limitations in Iran for these patients to freely access the desired medications, such as lower income or unavailable medications in the market. Such obstacles might induce both psychological distress and non-adherence trends, without any needed causal effect between the two.

While studies have mentioned the impacts of pre-transplant depression in non-adherence of liver and kidney transplant recipients (Scheel et al., 2018; Golfieri et al., 2019), they have suggested the positive impact of psychological interventions in adherence. It is also noticeable that in some studies, poor psychological scores before transplantation did not significantly modify patient adherence to immunosuppressive regimens when compared with psychologically stable patients (Michaud et al., 2016). This suggests further attention to considering the causal relationship between non-adherence and poor psychological scores. However, a major challenge in assessing post-transplant patients is the small size of study groups in many studies, including ours.

Our data suggest that the adherent patients have a higher overall QoL score, which is statistically significant in mental factors. Yet, differentiating QoL-affecting factors from adherence-affecting factors is not an easy task, as most of them such as age, the time elapsed after the transplantation, pre-transplant psychological status, and similar ones are common in both. Some of the prior studies indicate that the most adherent patients had lower complications such as organ rejection. Hence, they experience fewer re-hospitalizations, both in frequency and in duration, improving their QoL. Also, it has been reported that the patients who had spent more time on an organ waiting list had poorer QoL and adherence due to depression (Santos et al., 2012; Benzing et al., 2016). Our center is more focused on cadaver organs than living donors, which would result in more waiting time for most of the patients in this study, affecting the QoL and adherence after transplantation. This should be considered in further studies that these patients are more likely to suffer from post-transplant depression and lower QoL scores. What was shown in this study is that the only factor that significantly affected QoL total score has been income level. Although poor scores in multiple factors such as adherence, job status, re-hospitalization, and education level have resulted in lower overall QoL scores in the patients, the difference has not been statistically significant. Hence, it is challenging to draw the causal link between QoL and adherence as well. It is interesting that normally it is assumed that a higher patient survival rate as a result of higher adherence leads to higher QoL, which might not be true in all situations. In a study regarding HIV patients, it was surprisingly concluded that not always higher adherence would result in higher overall QoL (Geocze et al., 2010). The authors further explained that patients with the highest levels of adherence would suffer more from the side effects of their HAART medications. In other words, although these patients survived more than the average, their QoL was lower. The same conclusion may be expected in liver transplant patients as their immunosuppressive medication too can induce severe side effects as do HAART medications. However, such interpretations need to be further investigated in larger study groups considering multiple factors such as self-care, which was not included in our study. The authors suggested a more convenient medication regimen that induces less interference with daily activities would increase the QoL scores in HIV/AIDS patients, which can be generalized to other chronic conditions such as liver transplant recipients.

In conclusion, it seems that QoL is a multifactorial outcome, with such diverse and interacting affecting components. While our data suggest a statistically significant relationship between forgetfulness and income level with adherence and overall QoL scores, respectively, policymakers and researchers should not view these two factors in an oversimplified manner. In order to achieve a better QoL, there must be multiple support systems for the patients, including better access to suitable medications, psychological supports, educational devices, and a reliable system for the patients to ask for help.

Our results have to be thoroughly evaluated prior to being used in any policymaking, as this study is challenged by some limitations. Some studies have mentioned that using extended or sustained released forms of some immunosuppressive medications, such as Tacrolimus, can help improve patients’ adherence (Beckebaum et al., 2011). As this particular dosage form is not available in our country, it was impossible to assess its effects on patient adherence in this study. Another limitation of this study is the subject group size and it being single-center. The limited group size was due to the decrease in physical visits of the patients during COVID-19 lockouts. In order to establish a firm decision on the effects of QoL on adherence and *vice versa*, larger multi-central studies must be performed.

## Conclusion

Non-adherence is calculated to be 41% in the subjects of this study, for which the main influencing factors were forgetfulness, drug shortages, and economic difficulties for medication provision. The overall QoL score has been in all parameters higher in the adherent group compared with non-adherent patients. The difference in QoL was most meaningfully significant in mental composite score among other parameters of QoL.

## Data Availability

The raw data supporting the conclusions of this article will be made available by the authors, without undue reservation.
